# Factors Affecting the Patency of Radiocephalic Arteriovenous Fistulas Based on Clinico-Radiological Parameters

**DOI:** 10.7759/cureus.13678

**Published:** 2021-03-03

**Authors:** Kalesh Sadasivan, Usha Kunjuraman, Biju Murali, Induprabha Yadev, Ajayakumar Kochunarayanan

**Affiliations:** 1 Plastic and Reconstructive Surgery, Government Medical College Thiruvananthapuram, Thiruvananthapuram, IND; 2 Radiology, Government Medical College Thiruvananthapuram, Thiruvananthapuram, IND; 3 General Surgery, Government Medical College Thiruvananthapuram, Thiruvananthapuram, IND

**Keywords:** atherosclerosis, grading, haemodialysis, arteriovenous fistula, venous tap sign, vessel diameter, ultrasound

## Abstract

Arteriovenous fistulas are an important means of vascular access for long-term haemodialysis in patients with end-stage renal disease (ESRD). We evaluated the outcome of radiocephalic arteriovenous fistulas (RCAVFs) in 55 patients operated upon in our hospital in southern India. We studied the outcome of RCAVF surgery with the demographic factors, duration of diabetes, the diameter of the radial artery and cephalic vein, and any signs of atherosclerosis in the radial artery. We found that a small cephalic vein size of ≤ 2 mm, a negative cephalic vein tap test, a thickened, non-compressible, calcified radial artery on palpation, and evidence of atherosclerosis on radiological investigations were associated with a significant chance of RCAVF failure. A clinico-radiological grading of atherosclerosis for peripheral arteries is also proposed. Any patient presenting to the microsurgeon with a small cephalic vein size, a negative cephalic vein tap test, a thickened, non-compressible, calcified vessel on palpation, and tram-track calcification or whole vessel calcification or severely atherosclerotic vessel on radiological evaluation must be approached with caution regarding RCAVF creation and must be prepared for an arteriovenous fistula (AVF) creation at a higher level.

## Introduction

Arteriovenous fistulas are an important means of vascular access for long-term haemodialysis in patients with chronic renal failure with end-stage renal disease (ESRD) [1]. Autologous arteriovenous fistulas (AVF) are the first choice among other AVFs, as it is simple to perform, easily learned, has good patency rate, and fewer complications [2-3]. Radiocephalic AVF (RCAVF) was developed by Kenneth Charles Appell in 1965, and later, the first 14 cases were published by Brescia et al. in 1966 [4-5]. Distal RCAVF are the preferred means of vascular access in most parts of the world [6]. The greatest concern in any surgical AVF is its failure to mature. Fistula survival has been shown to be influenced by the institution where the surgery is performed [7], operative factors (e.g., greater intraoperative doses of heparin), vein diameters, type of surgical suturing, and perioperative factors, such as hypotension during dialysis [8-10]. In a meta-analysis, the prevalence of this primary fistula failure (PFF) in radiocephalic AVF had a pooled estimate of 15.3% (95% CI: 12.7 - 18.3%) [11]. It is known that 24% of RCAVFs thrombose directly after an operation or do not function adequately [12]. Although the radiocephalic type of arteriovenous fistulas are the first-line choices in most settings, meta-analysis has shown them to have lesser patency rates in the elderly compared to brachiocephalic AVF [13]. Flow-limiting stenosis develops in RCF earlier than in other upper limb fistulas (median: 113 days; 95% CI: 38 - 88) compared to brachiocephalic (BC) and transposed brachiobasilic (BB) AVF, but the time to fistula failure was not significantly different among them [14]. Maturation has been defined variously by ultrasound (e.g., having a blood flow rate greater than 500 mL/min), based on its structure/patency as fistulas with a diameter greater than 0.4 cm, or one that has successfully supplied adequate blood flow for hemodialysis [15]. Failure to use the created AVF inside its generally accepted maturation time of three months is defined as PFF [16]. In this paper, we intend to observe the outcomes of RCAVF surgeries done by the hybrid suture technique (see below) and elucidate the effect of arterial and venous vessel diameter, as determined by clinical examination, preoperative investigations, and intraoperative measurements, as a factor for its success.

## Materials and methods

Distal RCAVF, done at the proximal wrist level, is the preferred first-line method of AVF creation in chronic renal failure (CRF) patients in our institution. CRF is defined here as kidney damage characterised by albuminuria with albumin-to-creatinine ratio >30 mg/g in two of three spot urine specimens or glomerular filtration rate (GFR) < 60 mL/min/1.73 m^2^ for three months or more. We prospectively followed up 55 cases of RCAVF done in our plastic surgical unit in southern India since January 2014. Our surgical technique is the end-to-side anastomosis of the cephalic vein to a window created in the ventral wall radial artery, 5 cm proximal to the radial styloid. A hybrid (interrupted-continuous) suturing technique is used, where the posterior wall of the AVF is sutured with 7-0 or 8-0 polypropylene, in a continuous mode, and the anterior wall is sutured in an interrupted mode. The interrupted suturing method is time-consuming and the control of bleed or leak from the posterior surface is troublesome. This hybrid interrupted-continuous suturing technique has been found to increase the anastomotic compliance and reduce the narrowing than in the continuous method, especially in smaller vessels [10]. About 5 cm of the anastomosed vein adventitia is scored to allow for dilatation.

**Figure 1:**
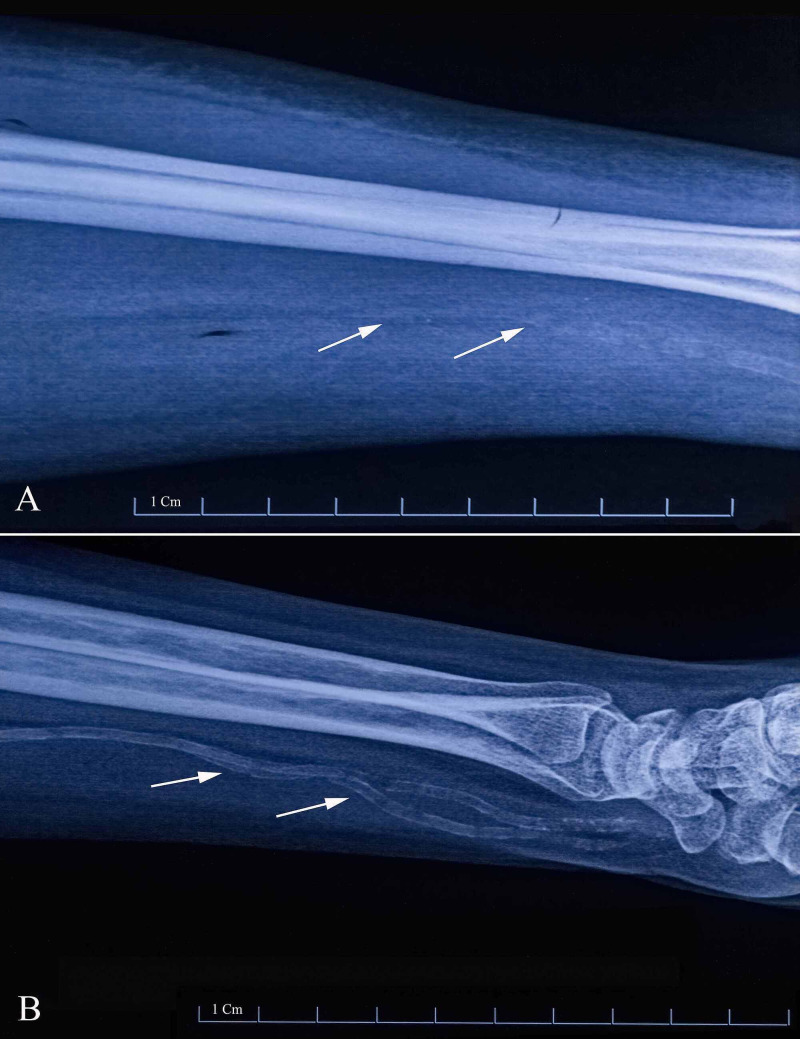
Representative radiographs of patients with different degrees of atherosclerosis (A) mild to moderate atherosclerosis; (B) severe atherosclerosis. Arrows point to the radial vessel wall calcification.

All patients in the current study underwent RCAVF by the hybrid technique. We looked into the details of renal failure, duration of diabetes, and other comorbidities, as well as demographic factors. Preoperative ultrasound scans (USS) were done to find the diameter of the radial artery and cephalic vein in millimetres, at a point 5 cm proximal to the radial styloid process in the distal forearm. The intraoperative measurement of the radial artery diameter and cephalic vein dilatation was also performed. The average diameter of the radial artery and cephalic vein, measured with preoperative USS and during the intraoperative assessment, was documented as the average vessel diameter (AVD). Digital x-ray films were taken to assess the presence or absence of atherosclerosis in the radial and ulnar arteries. Atherosclerosis was graded as mild-moderate (Figure 1A) or severe (Figure 1B), depending on the severity of findings in digital x-ray, ultrasound scans, a clinical observation by palpation of thickening of vessels, and by intraoperative assessment by direct observation (Table 1).

**Table 1:** Clinico-radiological Grading of Atherosclerosis of Peripheral Arteries *Presence of any one or more parameters will qualify the disease to be of the assigned stage

Grade	Clinical palpation	Digital x-ray	Ultrasound	Intraoperative
Grade 0: No atherosclerosis	Normal vessel	Normal	Normal	Normal
Grade I: Mild to moderate atherosclerosis	Thickened vessel but compressible^*^	Specks of calcification^*^	Mild diffuse atherosclerotic vessels^*^	Mild calcification with > 50% normal intervening areas for anastomosis^*^
Grade II: Severe atherosclerosis	Thickened, non-compressible, calcified vessel^*^	Tram track calcification or whole vessel calcification^*^	Severely atherosclerotic vessel^*^	Severely atherosclerotic vessels with < 50% normal areas for anastomosis^*^

To elicit the cephalic vein tap test, the patient was seated and the hand loosely hung by the side for 30 seconds so that the cephalic vein was naturally distended by gravity. The cephalic vein tap sign was defined as positive if a single finger percussion on the cephalic vein of the patient at the level of radial styloid was palpated by an examining finger on the course of the cephalic vein, placed 10 cm above the percussion point. This cephalic vein tap test was devised by the first author.

Postoperatively, the patients were followed up in subsequent visits at three and six months, as they came to the institution for haemodialysis. The success of the RCAVF was assessed as continued primary patency and being able to support haemodialysis at six months postoperatively with flow rates of 300 - 350 mL/min [17]. In this study, primary failure was defined as loss of primary patency during an assessment at three months or later with a blood flow non-supportive of haemodialysis at six months. 

## Results

A total of 55 patients underwent RCAVF in the department with a mean age of 46.49 ± 13.98 years. Of them, 20 were females (36.4%) and 35 were males (63.6%). The median duration of renal failure was 12 months (median interquartile range (IQR) = 6.00, 20.00]. The mean duration of disease for the patients was 14.96 ± 13.98 months. Fifteen patients had type 2 diabetes, and the mean duration of diabetes in them was 16.17 years. The preoperative tap sign in the cephalic vein at the wrist was positive in 47 cases (85.5%) and negative in eight cases (14.5%). With respect to patients with successful RCAVF who could be evaluated and fully followed up, 39 cases (90.7%) had a positive tap sign and four (9.3%) patients had a negative one. As per the classification of clinico-radiological grading of atherosclerosis in the forearm arteries used in the study, it was observed that 41 (74.55%) patients had Grade 0 atherosclerosis, 11 (20.0%) had Grade 1, and three (5.45%) had Grade 3 atherosclerosis (Table 1). The mean radial artery diameter was 2.07 ± 0.32 mm and that of the cephalic vein was 1.91 ± 0.39 mm. The evaluation of the patients who underwent RCAVF postoperatively showed that we had success in 42 (76.4%) and nine (16.4%) had fistula failure. Four patients (7.3%) expired before the evaluation could be made at six months and hence were excluded from the further analysis. In our study, diabetic patients constituted 15 (29.4%). Failure of the fistula occurred in nine (17.6%) patients.

Table 2 displays the various important factors associated with failure in patients with RCAVF as per the univariate analysis. While, age and sex of the patient were not important factors, a smaller cephalic vein, negative cephalic vein tap test, and grade 2 atherosclerosis were associated with a poor outcome. The success of the fistula was also significantly reduced with progressive grades of atherosclerosis. Compared to non-diabetic patients, diabetics have higher odds of fistula failure. Patients with a negative preoperative tap sign have a very high risk of fistula failure compared to a positive test. There is a statistically significant association between negative tap signs and failure (p-value = 0.002). 

**Table 2:** Important Factors Associated with Failure of RCAVF (n = 51) RCAVF: radiocephalic arteriovenous fistula

Factor	Attribute	RCAVF Success	RCAVF Failed	Odds Ratio (univariable)
Age	Mean age in years (standard deviation)	45.1 (14.6)	51.3 (6.6)	1.04 (0.98 - 1.11, p = 0.222)
Sex	Females with number of cases (percentage)	12 (75.0)	4 (25.0)	-
	Males with number of cases (percentage)	30 (85.7)	5 (14.3)	0.50 (0.11 - 2.32, p = 0.357)
Duration of diabetes (N = 15)	> 10 years	2.2 (5.7)	9.2 (9.3)	1.12 (1.03 - 1.24, p = 0.014)
Diabetes	Diabetic	9 (60.0)	6 (40.0)	-
	Non-diabetic	33 (91.7)	3 (8.3)	0.14 (0.02 - 0.62, p = 0.013)
Cephalic vein diameter (mm)	Mean diameter in mm (standard deviation)	1.9 (0.3)	1.5 (0.3)	0.03 (0.00 - 0.28, p = 0.005)
Cephalic vein tap test	Positive cases (percentage)	39 (90.7)	4 (9.3)	-
	Negative cases (percentage)	3 (37.5)	5 (62.5)	16.25 (2.98 - 109.71, p = 0.002)
Atherosclerosis	Number of Grade 0 cases (percentage)	33 (89.2)	4 (10.8)	-
	Number of Grade 1 cases (percentage)	8 (72.7)	3 (27.3)	3.09 (0.52 - 17.01, p = 0.189)
	Number of Grade 2 cases (percentage)	1 (33.3)	2 (66.7)	16.50 (1.31 - 408.16, p = 0.036)

Among males, 30 patients (85.7%) had successful RCAVF compared to 12 (75%) in females, but it failed to achieve statistical significance (p-value = 0.436; Fisher's exact test, two-sided). In patients with less than 50 years of age, 89.7% had success with fistula. However, only 7.2% of patients in the more than 50 years old group had a successful RCAVF; this failed to achieve statistical significance. Five (62.5%) of the patients with more than 10 years of diabetes had failure compared to one patient (14.3%) in those with a history of fewer than 10 years. This again did not achieve statistical significance (Fisher's exact test, two-sided; p-value = 0.119). With respect to atherosclerosis of the radial artery, there was an upward trend in the failure rate in various grades of atherosclerosis from 10% in grade 1 to 66% in grade 2, with a statistically significant p-value = 0.03 (p = 0.0429199, Fisher's exact test for count data). Eight percent of the patients with a radial artery diameter of more than 2 mm reported success, whereas only 9% had success with a size of less than 2 mm. However, this failed to achieve statistical significance (Fisher's exact test, two-sided; p-value = 0.663). Twenty-eight patients with a cephalic vein diameter over 2 mm and 14 patients with cephalic vein size less than 2 mm had successful RCAVF, which was statistically significant (Fisher’s exact test; p-value = 0.003).

## Discussion

The success of fistulas depends on various factors. The patient's age should not be a strict criterion for primary fistula creation [18], but one study has proven that age ≥ 65 years is a risk factor for AVF failure [1]. In our study, we found that the age of the patient did not emerge as a significant factor in the outcome of RCAVF. The female gender is at high risk for failure of an RCAVF, as evidenced in some works [6, 19-20], but we found no significant association. Women need extended time for an adequately matured AVF [21]. However, one study has expressed doubt regarding gender as a decisive factor [1].

Some works have established that the comorbidities are an increased risk for distal RCAVF failure. Diabetes was associated with delayed maturation and failure in RCVAF [19-20, 22]. However, these studies have not considered the duration of the illness, which is a major predictor of vessel involvement. To date, no studies have evaluated the duration of diabetes as a risk factor for a poor outcome in RCAVF. However, in this study, it was observed that although the absence of diabetes was a favourable factor in the success of AVF, the duration of diabetes of even more than 10 years was not a significant factor that had a risk of failure. This may be explained by the low percentage of diabetics in the sample. Peripheral vascular disease, hyperlipidaemia, and coronary artery disease (CAD) increase the risk of fistula failure [1]. It has been proven that obesity, previous vascular disease, increased high-sensitivity C-reactive protein levels, and optimal initial intraoperative blood flow (IOBF) < 190 ml/min are reasons for failed maturation [16]. Various studies have found that the vessel size of less than 2 mm is associated with higher failure rates [23-25], while others have a contrary opinion [11]. A cephalic vein diameter of 2 mm or less is a risk factor for failure [22, 26]. Radial artery diameter of ≤ 1.6 mm, cephalic vein diameter of ≤ 1.8 mm, and venous distension ≤ 0.4 mm are exact cut-off points, which best predict non-maturation of RCAVF [21]. A radial artery diameter of less than 2.5 mm was found to be significant in fistula failure [26]. A meta-analysis suggests that the critical diameter of the radial artery is 2 mm, and a cephalic vein diameter of 2 mm is needed for RCAVF success [27]. Patients with radial artery microcalcifications have non-favourable outcomes, and in the case of calcified vessels, brachial vessels are to be preferred [28]. Intraoperative low mean arterial pressure and absence of thrill in the immediate postoperative period have been proven as a factor for failure [11]. A cephalic vein diameter of 2 mm or less is a risk factor for failure [21-22, 26]. We had found that a cephalic vein of more than 2 mm was significantly associated with the success of RCAVF. Radial artery diameters ranging from ≤ 1.6 mm to 2.5 mm best predicted non-maturation of RCAVF in previous studies [21, 26-27], while in our study, no such association could be made out. No study to date has employed the clinically simple to perform cephalic vein tap test to predict the success of RCAVF. We found that a positive tap test is a very useful clinical sign associated with the success of RCAVF. Radial artery calcification and atherosclerosis had unfavourable outcomes in RCAVF [27]. Here, we graded peripheral arterial atherosclerosis into two grades and there was a significant chance of adverse results with increasing grades of atherosclerosis.

## Conclusions

The age and sex of the patient were not factors for the primary failure of an RCAVF in our study. A cephalic vein diameter of ≤ 2 mm, a negative cephalic vein tap sign, and atherosclerosis, especially Grade 2, were significantly associated with the failure of an RCAVF. In conclusion, any patient presenting to the plastic surgeon with a small cephalic vein size, a negative cephalic vein tap sign, and a thickened, non-compressible, calcified vessel on palpation, tram-track calcification, whole vessel calcification, or a severely atherosclerotic vessel on radiological evaluation must be approached with caution regarding RCAVF creation and must be prepared for an AVF creation at a higher level.
